# Effect of Position 1 Substituent and Configuration on APCI–MS Fragmentation of Norditerpenoid Alkaloids Including 1-*epi*-Condelphine

**DOI:** 10.1021/acsomega.2c05697

**Published:** 2022-10-25

**Authors:** Ashraf M. A. Qasem, Michael G. Rowan, Ian S. Blagbrough

**Affiliations:** School of Pharmacy, University of Bath, Bath BA2 7AY, U.K.

## Abstract

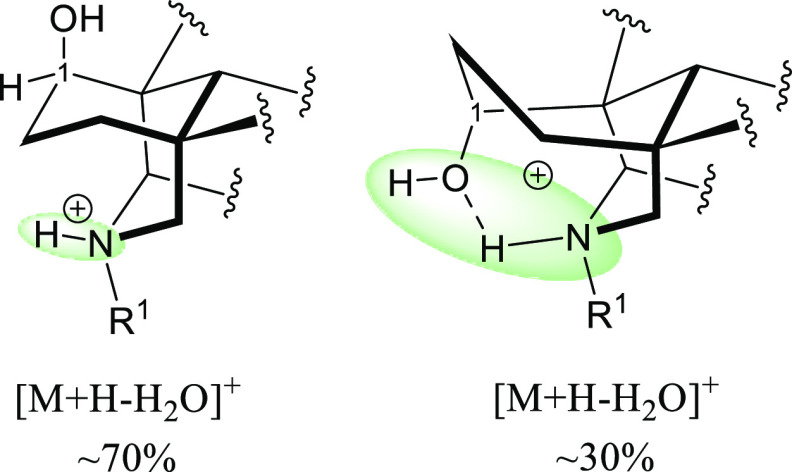

Norditerpenoid alkaloids (NDA) are hexacyclic highly oxygenated compounds, and the analysis of their 3D configuration is important as it helps to interpret their bioactive conformations. High-performance liquid chromatography/atmospheric pressure chemical ionization mass spectrometry (LC/MS–APCI) is a promising technique to investigate NDA stereochemistry. The effect of the alpha (α)-substituent at carbon 1 and its configuration on the stability of NDA in the mass spectrometer was studied. It was observed that 1-OH NDA are more stable compared to 1-OMe NDA due to the intramolecular H-bonding that exists in 1-OH NDA. In addition, 1-*epi*-condelphine **9** was found to be less stable in the mass spectrometer compared to condelphine **7** as the nitrogen is no longer hydrogen-bonded to the β-hydroxyl at position 1, which highlights the importance of the substituent configuration at carbon 1.

## Introduction

The structural investigation and elucidation of natural products is one of the major applications of mass spectrometry. The fragmentation pattern of the analytes is sensitive to the experimental conditions of ionization.^1^ Therefore, several analytical methods have been developed to control the amount of energy used to fragment the precursor ion with regard to the fragment ion.^1,2^ Several studies have been reported on the application of electrospray ionization tandem mass spectro-metry (ESI-MS/MS) to differentiate stereoisomers of different alkaloids groups such as indole alkaloids,^3^ indoloquinolizidine alkaloids,^4^ and matrine-type alkaloids.^5^ Electron impact-mass spectrometry (EI-MS) has been applied to study the structural effect on fragmentation, for example, to study the effect of stereoisomerism on EI fragmentation of eburnane-type alkaloids.^6^ In addition, EI-MS was applied to study the effect of the substituent configuration at position 1 of norditerpenoid alkaloids (NDA), where it was found that the intensity of the fragment peak [M – 15]^+^ was higher for 1-β-OH NDA compared to 1-α-OH NDA, whereas the intensity of [M-OH]^+^ was higher with 1-α-OH NDA compared to 1-β-OH NDA.^7,8^

There are only a few reports on the usage of high-performance liquid chromatography–atmospheric pressure chemical ionization mass spectrometry (HPLC–APCI–MS), which is a promising technique to investigate the effect of substituents’ configuration on the NDA skeleton fragmentation.^9−11^ APCI works on the analyte in the gas phase through nebulization (aerosol generation) by a high-speed gas and then desolvation of the droplets in the vaporization chamber. After that, ionization of the analyte happens in the gas phase through corona discharge, which is produced through a high-voltage needle. The CI reagent gas in APCI is the LC mobile phase (or analyte solvent) where the vaporized solvent forms several adduct ions through the reaction with electrons from corona discharge. In the positive-ion mode, proton transfer occurs from the adducts to the analyte. In the negative-ion mode, proton subtraction produces the molecular ion.^12^

The advantage of using APCI is that ionization occurs in the gaseous state than in the liquid state in ESI, which enables APCI to work with non-polar solvents. Also, APCI is less susceptible to matrix effects (including ion suppression) compared to ESI, and therefore, APCI can be considered for a wide range of applications including non-polar analytes.^11^ Although APCI uses high collision frequency which results in more fragments in the ionizer chamber compared to ESI (harder ionization),^13,14^ it is still considered a soft ionization method as the rapid desolvation reduces the thermal degradation considerably, which results in fewer fragmentations compared to hard ionization methods.^14^

**Figure 1:**
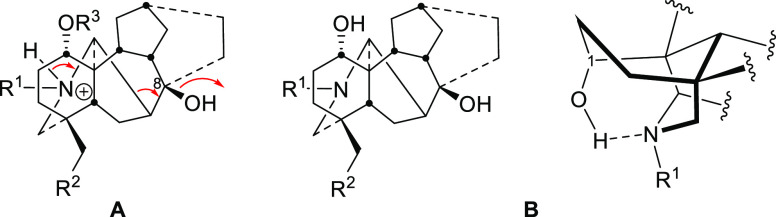
(A) Fragmentation of NDA skeleton at position 8 using APCI. (B) Intramolecular H-bond in 1-OH NDA where the secondary alcohol is axial.

It was demonstrated that using APCI ionization, the major fragmentation of the NDA skeleton occurs at position 8 where NDA with 8-OH, 8-OCH_3_, and 8-OAc fragment to show a loss of 18, 32, and 60 Da, respectively.^10^ It was also demonstrated using deuterium labeling that the fragmentation at position 8 starts from the nitrogen where it was shown that the deuterium atom introduced on the nitrogen atom is in the leaving fragment (loss of 20 Da with 8-OH NDA and loss of 62 Da with 8-OAc NDA).^10^Figure 1A shows the fragmentation at position 8 starting from the nitrogen atom.

The influence of substitution at position 1 was reported, where it was observed that the presence of α-OH at position 1 results in stabilization and lower fragmentation compared to 1-OMe NDA. It was proposed that the stabilization occurs due to intramolecular H-bonding (Figure 1B).^10^ In this study, the APCI fragmentation results of a series of 1-OMe and 1-OH NDA are reported, showing the effect of ring A conformation on the mass spectral fragmentation pattern, also highlighting the importance of the substituent configuration at position 1 on the stabilization of the NDA skeleton.

## Results and Discussion

### Effect of Carbon 1 Substituent on NDA Stability in APCI Mass Spectrometry

**Figure 2:**
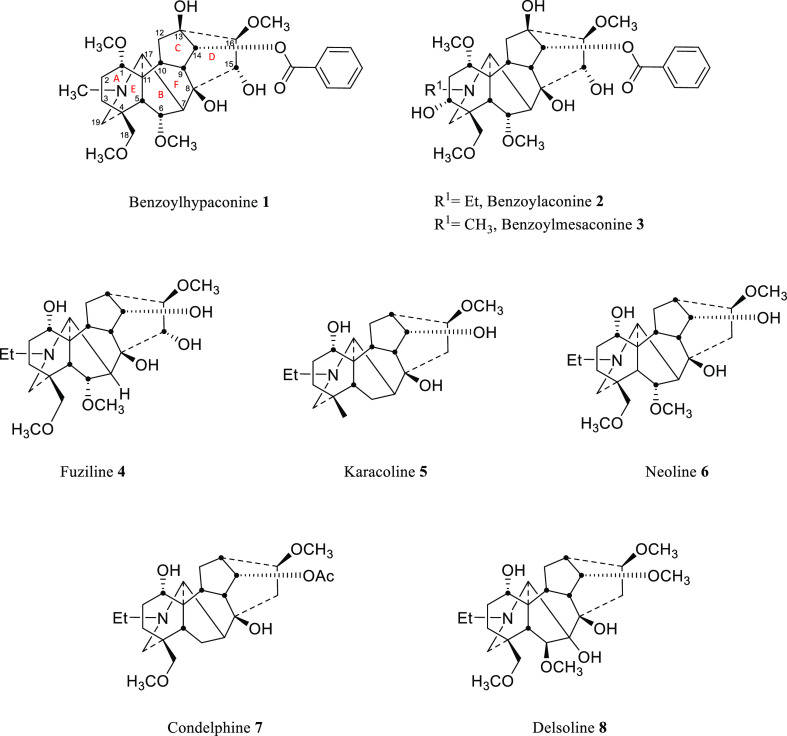
NDA **1–8**.

To study the effect of position 1 substituent on the stability of the NDA skeleton, eight compounds were chosen (Figure 2), where all of them possess a hydroxy group at position 8 (the initial fragmentation position). The APCI–MS method was applied to investigate the stereochemical effect of the carbon 1 substituent on the stability of alkaloids **1–8**. The APCI mass spectra were simple and showed the [M + H]^+^ parent ion alongside the major fragment ion [M + H – H_2_O]^+^. The detected signals and their relative abundance (*I*%) are given in Table 1.

**Table 1:** Detected Signal of the Parent Ion and Its Fragment Ion with Their Intensities (I %)

cmpd	molecular formula	[M + H]^+^*m*/*z*	[M + H]^+^*I*%	[M + H – H_2_O]^+^*m*/*z*	[M + H – H_2_O]^+^*I*%
**1**	C_31_H_43_NO_9_	574.3049	100	556.2936	57
**2**	C_31_H_43_NO_10_	590.3002	100	572.2781	62
**3**	C_32_H_45_NO_10_	604.3147	100	586.3016	58
**4**	C_24_H_39_NO_7_	454.2851	100	436.2730	30
**5**	C_22_H_35_NO_4_	378.2660	100	360.2551	29
**6**	C_24_H_39_NO_6_	438.2926	100	420.2817	27
**7**	C_25_H_39_NO_6_	450.2941	100	432.2811	31
**8**	C_25_H_41_NO_7_	468.3015	100	450.2894	33

**Figure 3:**
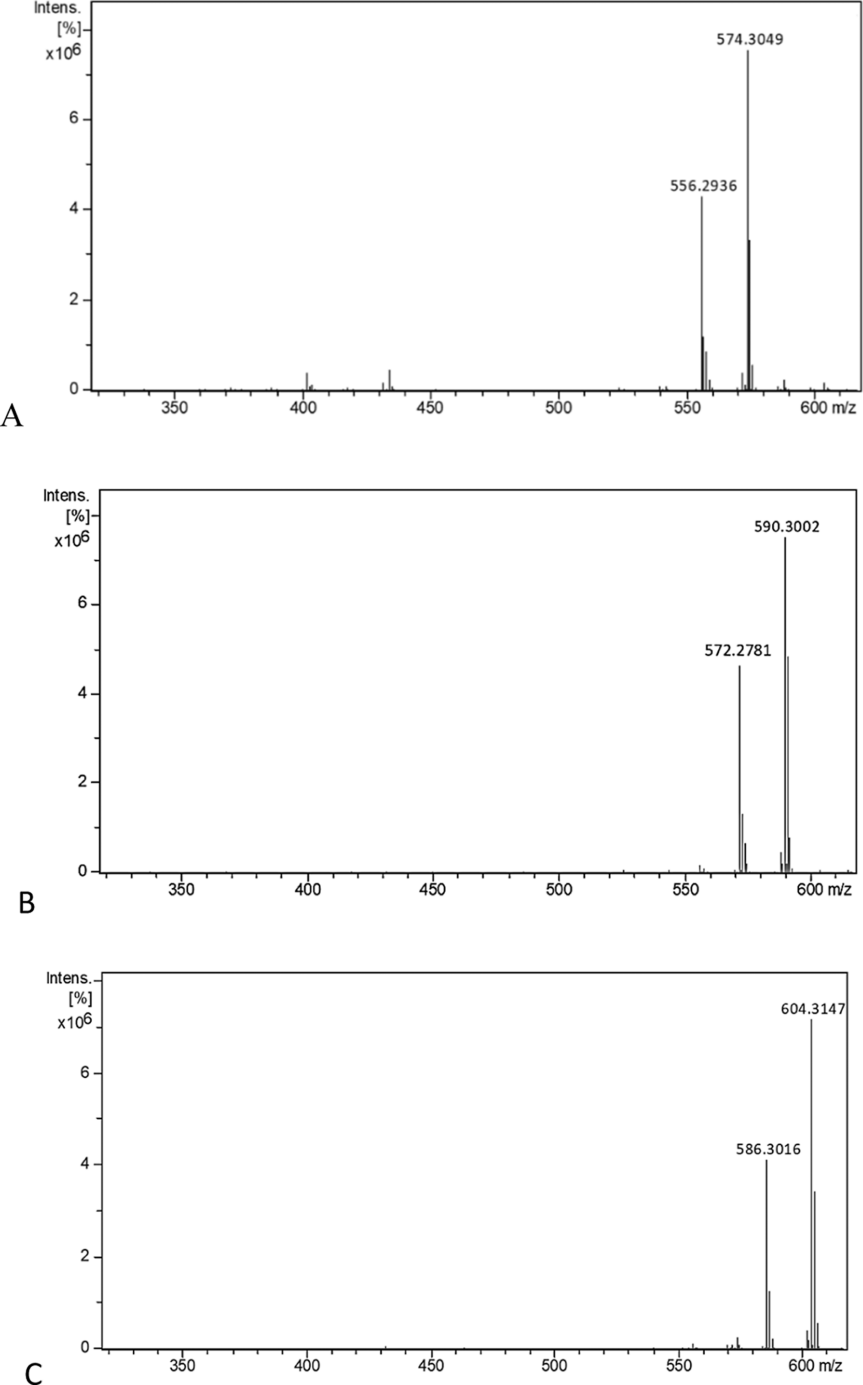
APCI mass spectra (A–C) of compounds **1**–**3**, respectively.

The APCI spectra of the 1-OMe alkaloids **1**–**3** were obtained and showed a parent [M + H]^+^ ion peak (100%) and a major fragment ion peak [M + H – H_2_O]^+^ (Table 1) at position 8 with a relative abundance of ∼60% (Figure 3).

**Figure 4:**
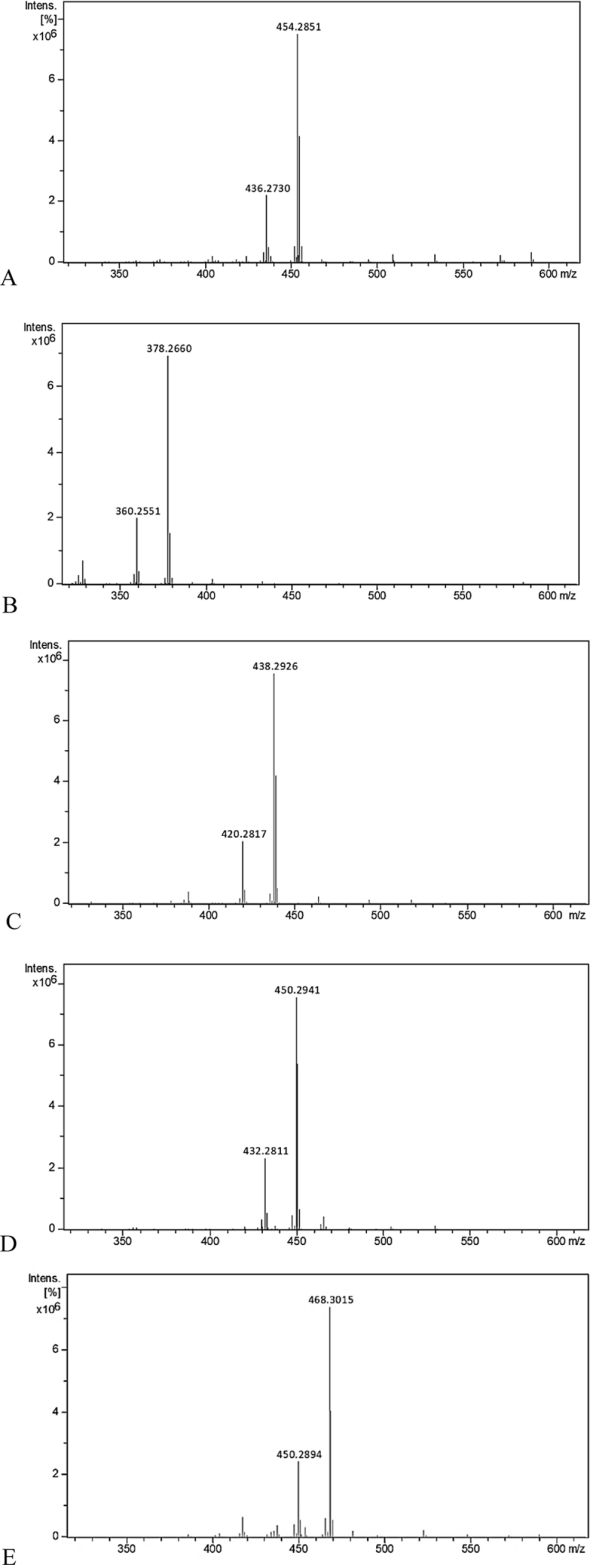
APCI mass spectra (A–E) of compounds **4–8**, respectively.

The obtained APCI mass spectra of compounds **4–8** were more stable as they showed less fragmentation at position 8 compared to compounds **1–3** (Figure 4). The intensity of the fragment ion peak observed for compounds **4–8** is around 30% compared to 60% for compounds **1–3**, which indicates the role of conformation in the stabilization of the NDA skeleton.

**Figure 5:**
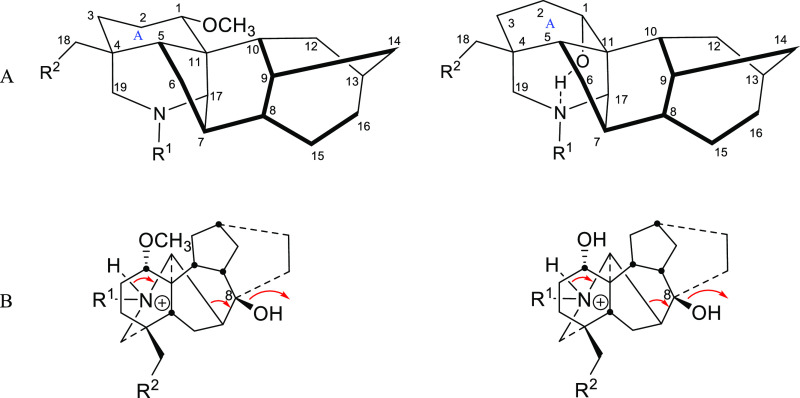
(A) 1-OMe and 1-OH NDA skeleton and (B) fragmentation of 1-OMe and 1-OH NDA at position 8.

**Figure 6:**
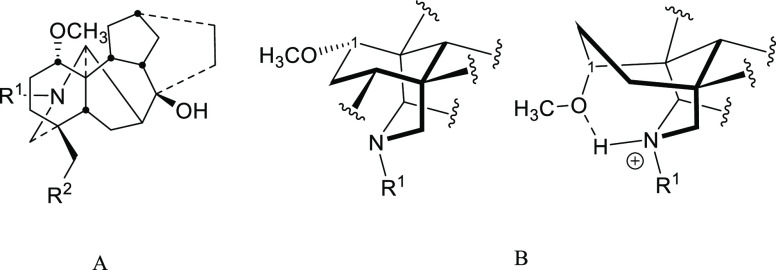
(A) 1-OMe NDA skeleton and (B) conformation of the AE bicycle as a free base (left) and salt (right).

We have reported that ring A conformations of 1-OMe and 1-OH NDA are different due to the intramolecular H-bond.^15^ The tertiary nitrogen is bonded (H-bond) to the 1-OH in compounds **4–8**, which results in flipping ring A into a boat conformation compared to a chair conformation in compounds **1–3** (Figure 5A). The explanation reported by Wada and co-workers^10^ for the stabilization of 1-OH NDA is that the introduced proton, which is the starting point of the fragmentation at position 8 (Figure 5B), is stabilized by an intramolecular hydrogen bond with 1-OH where ring A adopts a boat conformation.^15^ On the other hand, 1-OMe NDA salts also form intramolecular H-bonds between the methoxy group and the protonated nitrogen, which flips ring A from a chair conformation into a boat^16,17^ (Figure 6) and results in a stabilization effect similar to 1-OH NDA. Therefore, the reported theory does not explain the observed higher fragmentation of 1-OMe NDA compared to 1-OH NDA.

**Figure 7:**
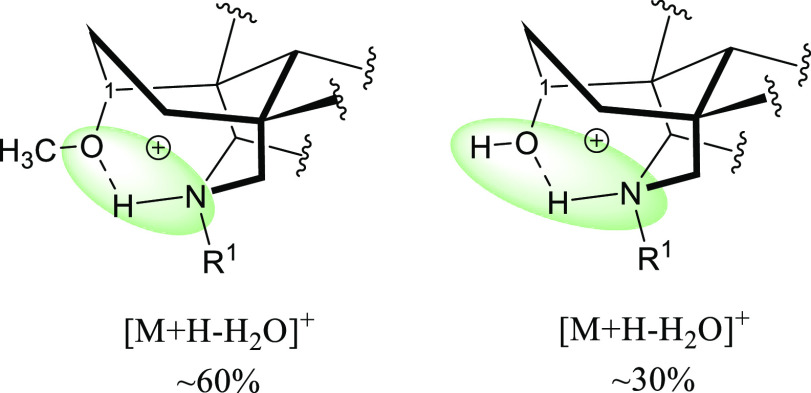
Charge delocalization (green area) in 1-OMe NDA salts (left) and 1-OH NDA salts (right).

A most reasonable explanation for the difference in the stability of 1-OH NDA and 1-OMe NDA is that the positive charge in 1-OH NDA is delocalized and stabilized over four atoms [N–H–O–H]^+^, while the positive charge in 1-OMe NDA is delocalized over three atoms [N–H–O]^+^, and therefore, 1-OMe NDA has higher tendency to lose the introduced proton on the nitrogen and consequently to lose H_2_O at position 8 (Figure 7).

### Effect of 1-OH Configuration on NDA Fragmentation

**Figure 8:**
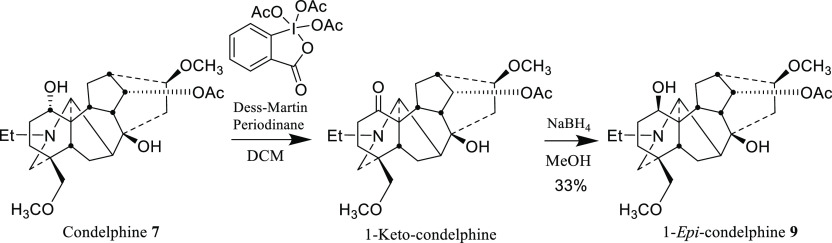
Oxidation of condelphine **7** and then reduction to obtain 1-*epi*-condelphine **9**.

The vast majority of NDA are functionalized with an α-substituent, and it was noted that a β-substituent should lead to less stabilization of the skeleton.^10,11^ To investigate the effect of the configuration on the NDA ring A conformation and skeleton MS fragmentation, condelphine **7** was converted into 1-*epi*-condelphine **9** (Figure 8).

**Figure 9:**
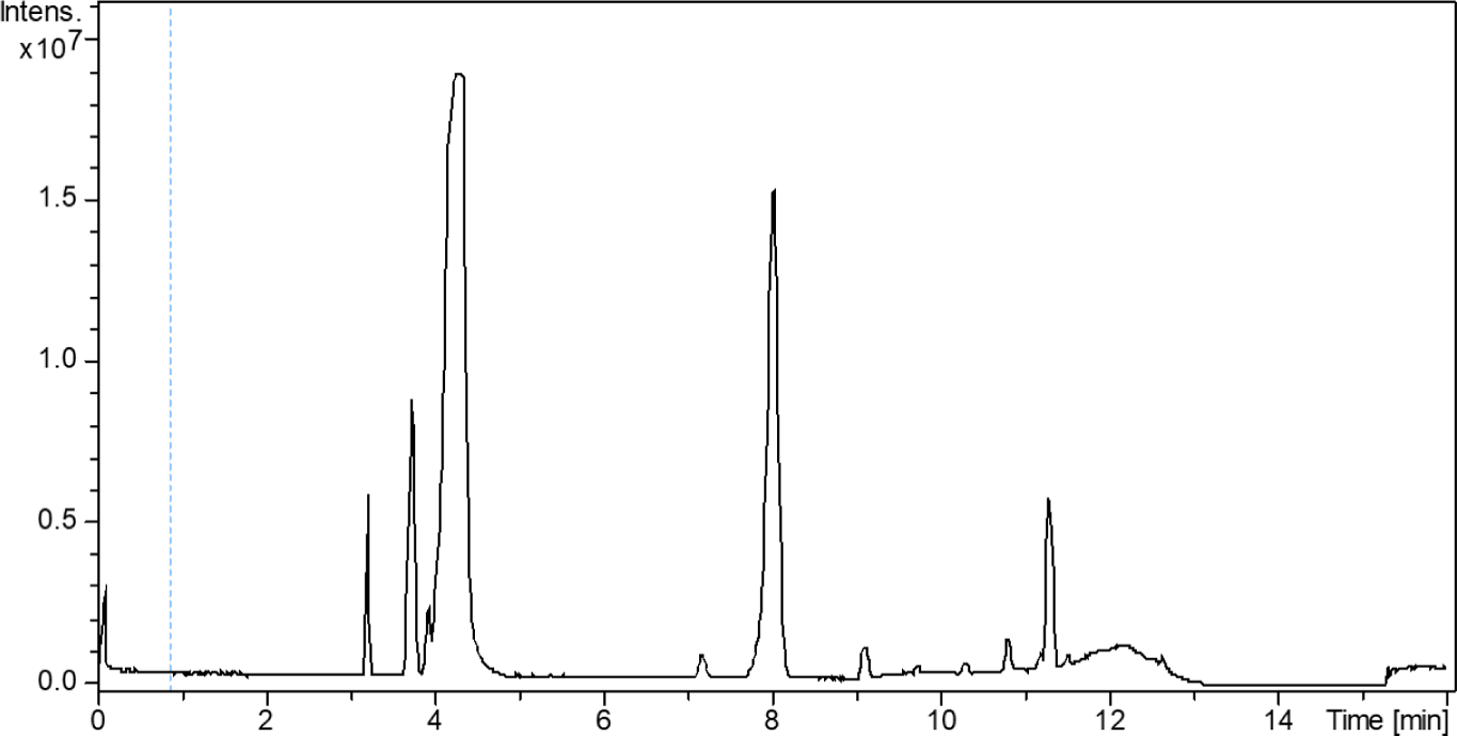
LC/MS–ESI ion chromatogram shows 1-*epi*-condelphine signal (4.3 min) and 1-keto-condelphine (8.0 min).

The first step of the reaction was oxidation of condelphine **7** (*R*_f_ = 0.28 in 10% MeOH/DCM) into 1-keto-condelphine (*R*_f_ = 0.35 in 10% MeOH/DCM) using 4 equiv of Dess–Martin periodinane in anhydrous dichloromethane at 20 °C for 24 h. 1-Keto-condelphine was then reduced with 1 equiv of NaBH_4_ to obtain 1-*epi*-condelphine **9** (33%). The final mixture was purified using an NX-C18 LC column, where 1-*epi*-condelphine **9** eluted after 4.3 min and 1-keto-condelphine at 8.0 min (Figure 9).

**Figure 10:**
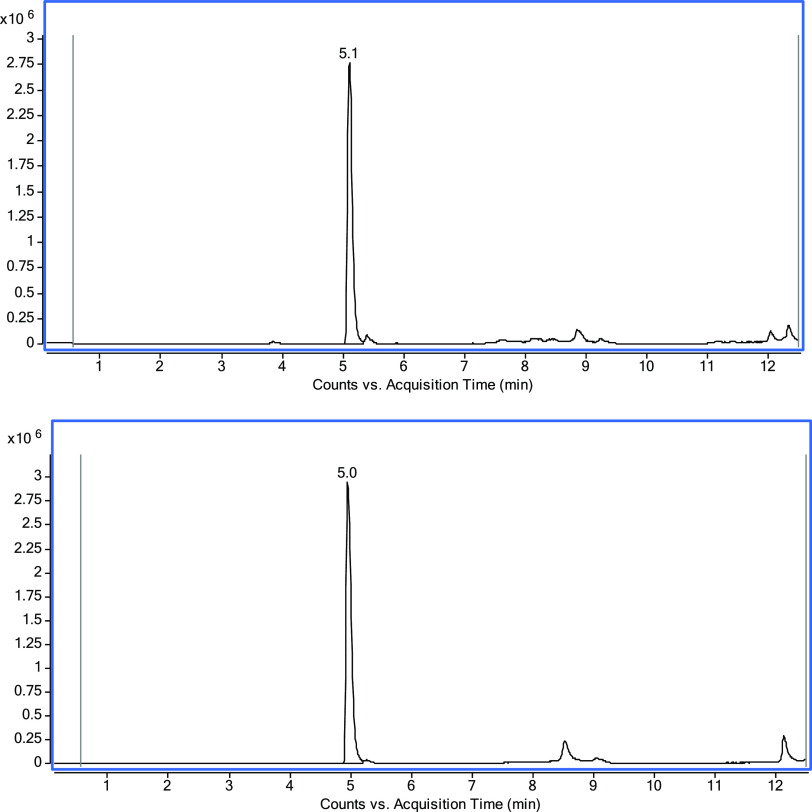
LC/MS–ESI ion chromatogram shows condelphine **7** elutes at 5.1 min (upper) and 1-*epi*-condelphine **9** elutes at 5.0 min (lower).

The obtained 1-*epi*-condelphine **9** showed TLC *R*_f_ = 0.32 (10% MeOH/DCM) compared to condelphine **7**, where the TLC *R*_f_ = 0.28 (10% MeOH/DCM). Both compounds were also analyzed using an analytical InfinityLab Poroshell 120 EC-C18 (3.0 × 50 mm, 2.7 μm) column, where condelphine **7** elutes at 5.1 min (Figure 10, upper), while 1-*epi*-condelphine **9** elutes at 5.0 min (Figure 10 lower). The reduction of 1-keto-NDA has also been reported using NaBH_4_,^18^ where both epimers were isolated, and 1-β-OH-NDA was the major epimer (3:1 ratio), which could be due to an effect from nitrogen where the delivery of the hydride complex is easier from the bottom face of the NDA skeleton. The obtained product from 1-keto-condelphine reduction was determined as 1-*epi*-condelpine **9**, and the product was not a mixture of epimers.

**Figure 11:**
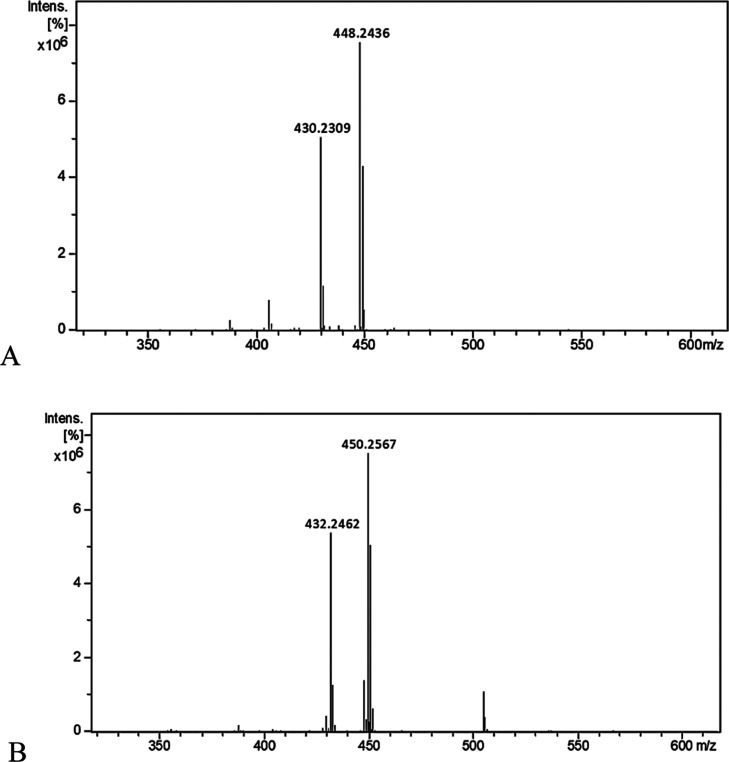
APCI mass spectra (A,B) for 1-keto-condelphine and 1-*epi*-condelphine, respectively.

The conformation of ring A of condelphine **7** was studied,^15^ and it was proved to be a twisted boat conformation due to the intramolecular H-bond with the basic nitrogen atom. Upon oxidation, the hydrogen bonding no longer exists, which is observed in the APCI mass spectrum where the intensity of the fragment peak was 67% compared to 31% for condelphine **7** (Figure 11A). After reduction, 1-*epi*-condelphine **9** shows a 72% fragment ion peak (Figure 11B). This is comparable to 1-keto-condelphine, which indicates that the skeleton is no longer stabilized by an intramolecular H-bond.

**Figure 12:**
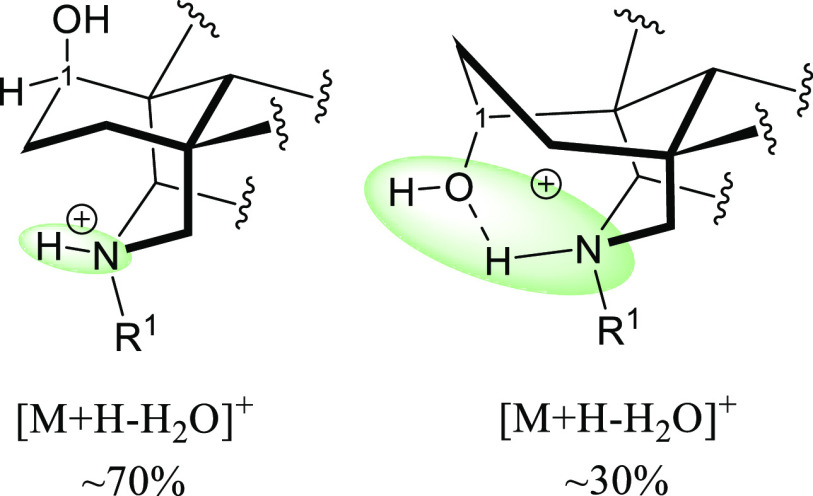
Charge delocalization (green area) in 1-β-OH NDA salts (left) and 1-α-OH NDA salts (right).

Figure 12 shows that the skeleton of 1-β-OH NDA is not stabilized by the intramolecular H-bond as the substituent at position 1 cannot form a H-bond with the nitrogen, and the positive charge is delocalized over two atoms compared to delocalization over four atoms in 1-α-OH NDA, which could be the reason for the observed difference in the APCI–MS fragmentation. NMR spectra also support the change of conformation as the chemical shift of the nitrogen in condelphine **7** is 54.2 ppm, while 1-*epi*-condelphine **9** has a lower chemical shift (43.8 ppm) for its nitrogen, which indicates that the nitrogen in 1-*epi*-condelphine **9** is no longer H-bonded to the 1-OH. The proton at position 1 in condelphine **7** resonates at 3.73 ppm, while in 1-*epi*-condelphine **9**, it resonates at 4.05 ppm, which indicates the change of ring A conformation where it was reported that the proton at position 1 has a higher chemical shift in 1-β-OH NDA compared to 1-α-OH NDA.^15^ Carbon 1 in condelphine **7** resonates at 72.2 ppm,^13^ while it resonates at 65.0 ppm in 1-*epi*-condelphine **9**, which is consistent with the ^13^C NMR spectral data of some NDA and their derivatives where such a decrease in the chemical shift of carbon 1 was observed when a 1-α-OH NDA was converted into the epimeric 1-β-OH NDA,^19,20^ for example, in the unusual epimerization of the 1-α-OH group in the NDA delphisine. Hydrolysis of delphisine in refluxing water afforded 14-*O*-acetyl-1-*epi*-neoline, and solvolysis of 8-*O*-acetylneoline in methanol afforded 8-*O*-methyl-1-*epi*-neoline. Likewise, solvolysis of delphisine in refluxing methanol afforded 8-*O*-deacetyl-8-*O*-methyl-1-*epi*-delphisine. For such an epimerization to occur, both α-1-OH and 8-OAc functional groups are necessary.^20^

## Conclusions

The stability of the NDA skeleton was studied using APCI–MS, where it was observed that the alpha (α)-substituent at carbon 1 affects the stability of the NDA towards fragmentation. It was found that 1-OH NDA are more stable compared to 1-OMe NDA, which showed higher intensity of the fragment-ion peak. That difference in stability could be due to the charge delocalization where the positive charge delocalizes in 1-OH NDA over four atoms compared to three atoms in 1-OMe NDA, which decreases the chance of the proton transfer in the fragmentation scheme. In addition, the effect of carbon 1 substituent configuration was studied by the synthesis of 1-*epi*-condelphine **9**, where it was found that it is less stable in the mass spectrometer compared to condelphine **7** as the nitrogen is no longer bonded to the β-hydroxyl at position 1. The application of APCI–MS is a promising technique to study the 3D configuration of NDA as it leads to a better understanding of their possible 3D-conformations in biological fluids.

## Experimental Section

### Materials and General Methods

Fuziline **4**, condelphine **7**, and delsoline (belsoline) **8** were donated by Carbosynth Ltd. (UK). Benzoylhypaconine **1**, benzoylmesaconine **2**, benzoylaconine **3**, and neoline **6** were purchased from Carbosynth Ltd. (UK). Karacoline **5** was purchased from Latoxan (France). All other chemicals were purchased from Sigma-Aldrich (UK) and used as received. Chloroform-*d* (99.8% D atom, CDCl_3_) was used for NMR experiments purchased from Cambridge Isotope Laboratories (USA). All other solvents were of HPLC grade, ≥99.9% purity (Fisher Scientific, UK, and VWR, UK).

### Instrumentation

Analytical thin-layer chromatography was performed using aluminum backed sheets of precoated silica gel (Merck Kieselgel 60 F254). Compounds were visualized by staining with iodine vapor, and Dragendorff solution, stock solution, was prepared by mixing bismuth subnitrate (1.7 g) with water (80 mL) and glacial acetic acid (20 mL). Aqueous potassium iodide solution (50% w/v, 100 mL) was then added and stirred until dissolved. The solution was stored in an amber bottle. The working solution was prepared by mixing the stock solution (100 mL) with glacial acetic acid (200 mL), made up to volume (1 L) with distilled water, and stored in an amber bottle. The purification of 1-*epi*-condelphine from 1-keto-condelphine was done using Gemini 5 μm NX-C18 110 Å, LC Column 250 × 10 mm with MaXis HD quadrupole electrospray time-of-flight (ESI-QTOF) mass spectrometric detection (Bruker Daltonik GmbH, Bremen, Germany). The comparison between condelphine and 1-*epi*-condelphine was done using LC–MS analyses performed using an Agilent QTOF 6545 with a Jetstream ESI spray source coupled to an Agilent 1260 Infinity II Quat pump HPLC with a 1260 autosampler, a column oven compartment, and a variable wavelength detector (VWD). The MS was operated in the positive-ion mode with the gas temperature at 250 °C, the drying gas at 12 L/min, and the nebulizer gas at 45 psi (3.10 bar). The sheath gas temperature and flow were set to 350 °C and 12 L/min, respectively. The MS was calibrated using a reference calibrant introduced from the independent ESI reference sprayer. The VCap, Fragmentor, and Skimmer were set to 3500, 100, and 45, respectively. Chromatographic separation of a 5 μL sample injection was performed on a InfinityLab Poroshell 120 EC-C18 (3.0 × 50 mm, 2.7 μm) column using H_2_O (Merck, LC–MS grade) with 0.1% formic acid (FA, Fluka) v/v and methanol (MeOH, VWR, HiPerSolv) with 0.1% FA v/v as mobile phases A and B, respectively. The column was operated at a flow rate of 0.3 mL/min at 40 °C starting with 1% mobile phase B for 3 min; thereafter, the gradient was initiated and run for 2 min to a final 100% B, held at 100% B for 3 min and then returned to 1% B, and held for re-equilibration for 3.9 min in a total run time of 12 min. The VWD was set to collect 254 and 320 nm wavelengths at 2.5 Hz. Data processing was automated in a Qual B 07.00 with a Find by formula matching tolerance of 10 ppm. ^1^H NMR spectra were recorded with a Bruker AVANCE III (500 MHz) spectrometer at 25 °C. Chemical shifts are given in parts per million (ppm) referenced to the CDCl_3_ solvent or its residual CHCl_3_ signal and reported as chemical shift (δ), multiplicity (br = broad, d = doublet, dd = doublet of doublet, m = multiplet, s = singlet, and t = triplet), coupling constant (*J* absolute values and rationalized to 1 d.p. in Hz), relative integral, and assignment. ^1^H–^15^N HMBC spectra were recorded on a Bruker AVANCE III (^15^N Larmor precession frequency 50.67 MHz) spectrometer at 25 °C. The spectra were externally calibrated with a MeNO_2_ solution (50% in CDCl_3_, v/v), recorded, and set at δ_N_ 379.8 ppm, and the correction factor was measured as −511.72 on our spectrometer. High-resolution time-of-flight (HR TOF) mass spectra were obtained on a Bruker Daltonics “micrOTOF” mass spectrometer using electrospray ionization (ESI) (loop injection +ve ion mode).

### APCI–MS Conditions

Accurate mass spectrometry analyses were conducted using a MaXis HD quadrupole electrospray time-of-flight (APCI-QTOF) mass spectrometer (Bruker Daltonik GmbH, Bremen, Germany), using a glass syringe (Hamilton) and a syringe pump (KD Scientific, Model 781100) for infusions at a flow rate of 3 μL/min. Analyses were performed in the APCI positive-ion mode with the capillary voltage set to 4500 V, corona discharge 4000 V, nebulizing gas at 0.4 bar, APCI temperature was set at 280 °C, and drying gas temperature at 240 °C. The TOF scan range was from 50 to 1000 mass-to-charge ratio (*m*/*z*). The MS instrument was calibrated using an APCI tuning solution (Sigma-Aldrich, U.K.). All samples were prepared in isopropanol at 20 μg/mL. Data processing was performed using the Compass Data Analysis software version 4.3 (Bruker Daltonik GmbH, Bremen, Germany).

### Synthesis of 1-*epi*-Condelphine **9**

Condelphine **7** (*R*_f_ = 0.28 in 10% MeOH/DCM) (0.0134 mmol, 6 mg) was dissolved in anhydrous dichloromethane (10 mL) under anhydrous N_2_ gas, and Dess–Martin periodinane (4 equiv, 0.0535 mmol, 22.7 mg) was added. The reaction mixture was stirred at 20 ^o^C for 24 h and then concentrated under vacuum, and the product, 1-keto-condelphine, was used in the next step without purification. The crude 1-keto-condelphine (*R*_f_ = 0.35 in 10% MeOH/DCM) was reduced using sodium borohydride (0.013 mmol, 0.5 mg) in anhydrous methanol (5 mL) at 20 ^o^C for 24 h. The mixture was then concentrated under vacuum and purified using HPLC to obtain the title compound **9** (2 mg, 33%) as a pale yellow oil. *R*_f_ = 0.32 (10% MeOH in dichloromethane). HRMS (ESI): *m*/*z* calcd for C_25_H_40_NO_6_, 450.2856; found, 450.2836 [M + H]^+^. ^1^H NMR (500 MHz, CDCl_3_): δ (ppm) (including) = 1.13 (br t, 3H, NCH_2_**CH**_**3**_), 2.06 (s, 3H, COCH_3_), 2.06–2.13 (m, 1H, H19_ax_), 2.30–2.35 (m, 1H, H19_eq_), 2.43–2.57 (m, 2H, N**CH**_**2**_CH_3_), 2.64 (dd, *J* = 7.9, 4.8, 1H, H13), 2.76 (br s, 1H, H17), 3.01 (d, *J* = 8.8, 1H, H18A), 3.15 (d, *J* = 8.8, 1H, H18B), 3.27 (s, 3H, 16-OCH_3_), 3.28–3.31 (m, 1H, H16), 3.32 (s, 3H, 18-OCH_3_), 4.05 (t, *J* = 7.0, 1H, H1), 4.87 (t, *J* = 4.9, 1H, H14). ^13^C NMR (125 MHz, CDCl_3_): δ (ppm) (including) = 13.3 (NCH_2_**CH**_**3**_), 21.1 (CO**CH**_**3**_), 36.4 (C13), 48.1 (N**CH**_**2**_CH_3_), 56.0 (16-OCH_3_), 56.5 (C19), 59.5 (18-OCH_3_), 63.5 (C17), 65.0 (C1), 76.9 (C14), 78.9 (C18), 82.0 (C16).
